# Screening of Peptide Libraries against Protective Antigen of *Bacillus anthracis* in a Disposable Microfluidic Cartridge

**DOI:** 10.1371/journal.pone.0026925

**Published:** 2011-11-28

**Authors:** Joshua M. Kogot, Yanting Zhang, Stephen J. Moore, Paul Pagano, Dimitra N. Stratis-Cullum, David Chang-Yen, Marek Turewicz, Paul M. Pellegrino, Andre de Fusco, H. Tom Soh, Nancy E. Stagliano

**Affiliations:** 1 United States Army Research Laboratory, Sensors and Electron Devices Directorate, Adelphi, Maryland, United States of America; 2 Cynvenio Biosystems, Inc., Westlake Village, California, United States of America; 3 CytomX Therapeutics, LLC, Santa Barbara, California, United States of America; 4 Department of Mechanical Engineering, University of California Santa Barbara, Santa Barbara, California, United States of America; University of Crete, Greece

## Abstract

Bacterial surface peptide display has gained popularity as a method of affinity reagent generation for a wide variety of applications ranging from drug discovery to pathogen detection. In order to isolate the bacterial clones that express peptides with high affinities to the target molecule, multiple rounds of manual magnetic activated cell sorting (MACS) followed by multiple rounds of fluorescence activated cell sorting (FACS) are conventionally used. Although such manual methods are effective, alternative means of library screening which improve the reproducibility, reduce the cost, reduce cross contamination, and minimize exposure to hazardous target materials are highly desired for practical application. Toward this end, we report the first semi-automated system demonstrating the potential for screening bacterially displayed peptides using disposable microfluidic cartridges. The Micro-Magnetic Separation platform (MMS) is capable of screening a bacterial library containing 3×10^10^ members in 15 minutes and requires minimal operator training. Using this system, we report the isolation of twenty-four distinct peptide ligands that bind to the protective antigen (PA) of *Bacilus anthracis* in three rounds of selection. A consensus motif **WXCFTC** was found using the MMS and was also found in one of the PA binders isolated by the conventional MACS/FACS approach. We compared MMS and MACS rare cell recovery over cell populations ranging from 0.1% to 0.0000001% and found that both magnetic sorting methods could recover cells down to 0.0000001% initial cell population, with the MMS having overall lower standard deviation of cell recovery. We believe the MMS system offers a compelling approach towards highly efficient, semi-automated screening of molecular libraries that is at least equal to manual magnetic sorting methods and produced, for the first time, 15-mer peptide binders to PA protein that exhibit better affinity and specificity than peptides isolated using conventional MACS/FACS.

## Introduction

Affinity reagents are molecular recognition elements (MREs) that specifically bind to their targets with high affinity. Thus, their effectiveness constitutes the first and the most important step in pathogen detection and response. Hybridoma monoclonal antibody generation technology has been the most common method for isolating affinity reagents for more than 30 years. However, hybridoma technology requires significant time, cost, and resources [1], [2]. As a result, the demand for high performance affinity reagents for novel molecular targets outpaces the current technology. Currently, a number of synthetic alternatives to hybridoma technology are under development including mRNA and ribosome display [3], eukaryotic virus display [4], [5], and bacterial and yeast surface display [6], [7] to more rapidly generate affinity reagents that can be used for diagnostics, proteomics, and therapeutic applications [8], [9].

When considering the desire to automate the selection process coupled with the overall time required to develop new recognition binders against a target of interest, the bacterial display is uniquely advantageous. The bacterial display technology offers an alternate strategy for generating tailor-made affinity ligands in a short time period (e.g., days to weeks), since one round of selection or screening can be performed in one day with bacterial cells [6], [10]. In this method, cellular machinery is used to generate billions of diverse polypeptide molecules that can be screened with high throughput methods to identify unique polypeptide sequences for a desired target [10]. Briefly, the fifteen amino acid, random polypeptide sequences are displayed on the surface of the *E.coli* during arabinose induction on a circularly permutated derivative of the outer membrane protein, OmpX, referred to as eCPX [11], [12]. The eCPX enables better peptide display off of the membrane surface, and is a biterminal display scaffold, displaying both the random peptide as a flexible linear sequence at the N-terminus and an expression tag sequence at the C-terminus for expression normalization [12]. Bacterial display libraries using either the OmpX or eCPX have been used previously to isolate polypeptide binding reagents to streptavidin [12], vascular endothelial growth factor (VEGF) [13], adult neural stem cells [14], protease activated pro-domains [15], and classification of breast tumor subtypes [16].

To isolate the bacterial clones which express peptide sequences with high affinity to the target, conventional approaches require multiple rounds (often three sorting rounds) of magnetic separation for pre-enrichment followed by fluorescence activated cell-sorting (FACS). FACS sorting is limited to at most 10^8^ cells in one session, whereas magnetic sorting can accommodate 10^9^ to 10^10^ clones per sort with more rapid results and greater recovery [17], [18], [19], [20], [21], [22]. Although this hybrid approach has proven to be effective over manual magnetic sorting, it is labor-intensive, and the sorting results are known to be operator-dependent [23]. Furthermore, the high capital and maintenance cost of FACS instruments limit its accessibility. Another limitation of FACS for both medical and DoD applications is the potential for generating an aerosolized biohazard at the nozzle when dealing with infectious pathogens; additional steps need to be taken to reduce this hazard, such as adding an aerosol management unit, further increasing cost.

**Figure 1:**
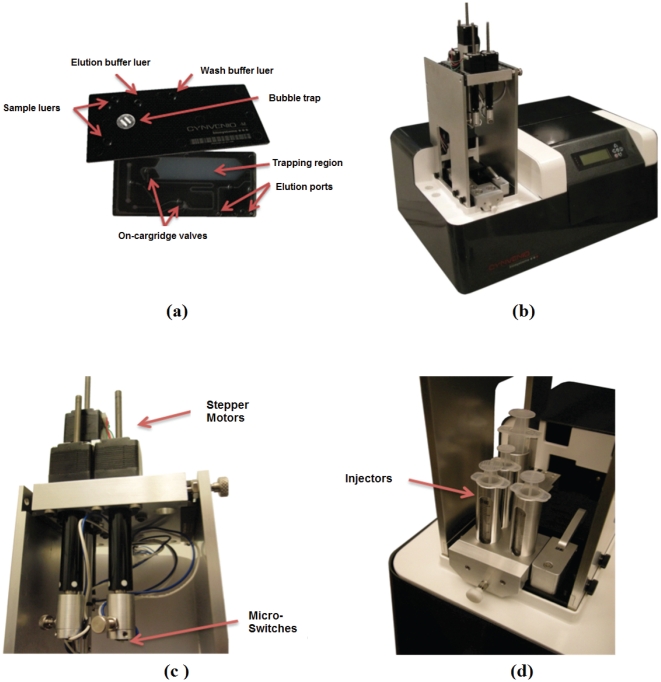
MMS Platform. A) MMS disposable cartridge with front side showing sample luer interfaces and backside showing separation region, on-cartridge valves and fluid path ways; B) automated MMS instrument with a volumetric control module for precise fluid injection speeds and volumes; C) Stepper motors are implemented to actuate injectors with micro-switches for injector location sensing; D) Off-shelf syringes are used as injectors for volumetric sample injection.

**Figure 2:**
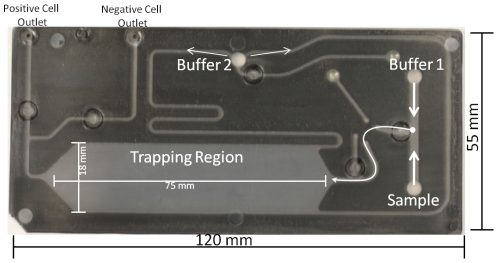
MMS Disposable Cartridge. The disposable MMS cartridge is made from polypropylene with outer dimensions of 120 mm×55 mm. The sample is mixed with buffer 1 prior to entering the trapping region. Buffer 2 is used to wash away all of the cells not bound to magnetic beads in the trapping region, which are collected at the negative cell outlet. Buffer 2 is also used to elute the cells trapped on the magnetic beads into the positive cell outlet for overnight growth for subsequent sorting or analysis of the enrinched population.

To address the need for a rapid, safe, efficient, cost effective, and reproducible affinity ligand selection, we have developed a semi-automated magnetic bacterial cell sorting system, the micromagnetic cell sorter (MMS), equipped with disposable microfluidic cartridges (Figures 1, 2). As an alternative to glass MMS cartridges [24] these low cost , highly reproducible and disposable polypropylene cartridges are autoclavable and limit any aerosolization of potential biohazards during library sorting, since all the fluid management, mixing, and sorting is accomplished within the card. The ability to perform semi-automated sorting in a disposable, self-contained microfluidic cartridge with reproducible results is critical for the DoD since any new defense threats could be safely sorted in a native state ahead of any available recombinant form.

In order to evaluate the potential of a platform for bacterial library sorting, there are several key capabilities that must be considered. For example, high throughput screening is desired to handle large libraries (several mL of >10^10^ member libraries) in a reasonable time frame (several minutes). Without rapid throughput, practical application will be difficult. Typically with the currently employed sorting technologies relying on a combination of MACS and FACS sorting, the throughput is determined by the MACS sample pre-enrichment prior to FACS sorting since fluorescence cell sorting methods using ultra high-speed sorting only approach 100,000 cells/sec [25] .

The recovery or fraction of binders collected relative to the total number of binders in the naïve library is extremely critical to not only affinity ligand development but also applications in medicine for cell identification, such as cancer cell isolation and population enrichment [26], [27], [28]. To assess recoverability, experiments were conducted on the MMS system to determine the rare cell and ultra-rare cell recovery (populations less than 0.001%) capability of the instrument compared to manual MACS [25], [26]. The automated MMS platform presented herein is designed for greater consistency and reliability compared to the manual MACS.

To demonstrate the sorting ability of the MMS system to isolate peptide binders, protective antigen (PA) protein of *Bacillus anthracis* was chosen for evaluation. The eCPX (CytomX Therapeutics; San Francisco, CA) bacterial display library [11], expressing ∼3×10^10^ discreet random 15 amino acid peptides, was screened for affinity reagents capable of binding to PA. Of note, comparable results to manual MACS and FACS screening were obtained and are presented herein. In addition, excellent recovery performance through MMS selection yields a consensus sequence among 24 unique binders and directly correlates to the best MACS/FACS binder sequence. The affinity of several clones were characterized using flow cytometry analysis to investigate the range of binding affinity in products using both conventional and MMS isolation.

## Results

**Figure 3:**
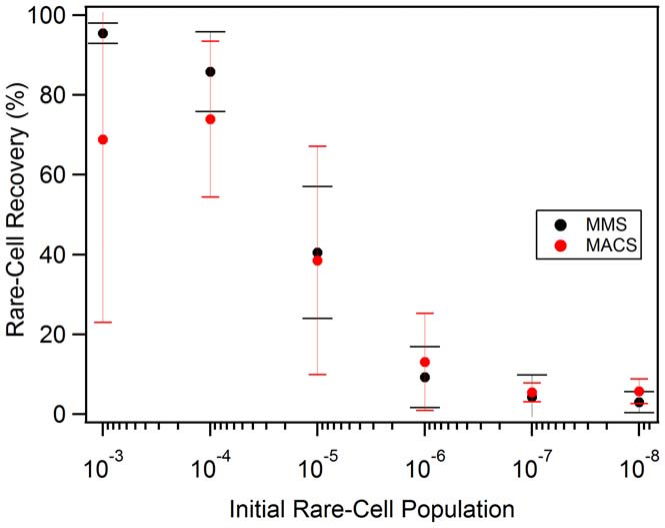
Rare-cell and Ultra-rare Cell Recovery Comparison. Rare-cell and ultra-rare cell recovery results for the MMS (black) and manual MACS (red) comparison. In four independent trials, both the MMS and MACS show cell recovery down to 10^−8^, with the MMS having greater between experiment cell recovery performance through all of the populations tested, given the smaller RMSD reported as error bars for each sample.

The MMS exhibited better overall rare-cell recovery compared to manual MACS when evaluating the reproducibility of the rare-cell recovery in each method, as noted by the greater between-sample variance in the manual MACS samples in four independent trials, shown in Figure 3. The MMS had equivalent rare-cell recovery within experimental error with specific percentage at 10^−3^ (95% to 65% recovery), 10^−4^ (85% to 75% recovery), and 10^−5^ (40% to 38% recovery) of the initial rare-cell population and nearly equivalent rare-cell recovery at 10^−6^ (15% to 15%), 10^−7^ (5% to 5%), and 10^−8^ (1% to 3%) (Fig. 3). The MMS had a lower overall average RMSD compared to manual MACS, 7.47% and 18.6% respectively. Both MMS and manual MACS demonstrated the capability for ultra-rare cell recovery of at initial rare-cell populations as low as 10^−8^ (Fig. 3). Statistical analysis of the MMS and MACS recovery at each rare cell population was performed using a Wilcoxon Matched-Pairs Signed-Ranks Test since the outcome of both sorting methods was dependent on the initial spiked-sample. The within population results (for 10^−3^ to 10^−8^) for the MMS and MACS comparison suggested no statistical difference between the methods. Only the 10^−4^ (p-value = 0.125) had differences approaching the 90% confidence limit with the small sample size tested (N = 4). Analysis of the entire sample (N = 24) resulted in a p-value = 0.3104, further confirming there was no statistical difference between the magnetic sorting methods.

**Figure 4:**
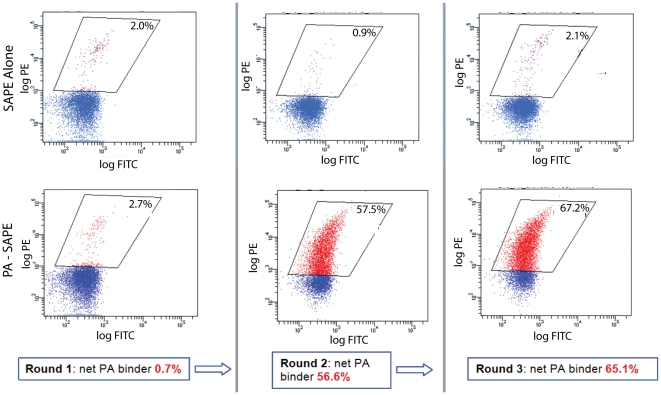
Flow Cytometry Analysis of MMS Results per Sorting Round. Flow cytometry analysis of the fraction of target-binding clones in the enriched population after incubation with Streptavidin-R-Phycoerythrin (SAPE) fluorescently labeled biotinylated PA protein target. The intensity of PE fluorescence (*y*-axis) represents the level of binding on the cell surface; this may be due to either a high expression or a high affinity for the target. Following one round of MMS, 0.7% (net) of the population exhibited PA binding peptides. Following two rounds of MMS, 56.5% (net) of the population exhibited target-binding peptides. And 65.1% (net) of the population exhibited target-binding peptides after three rounds MMS selection.

**Figure 5:**
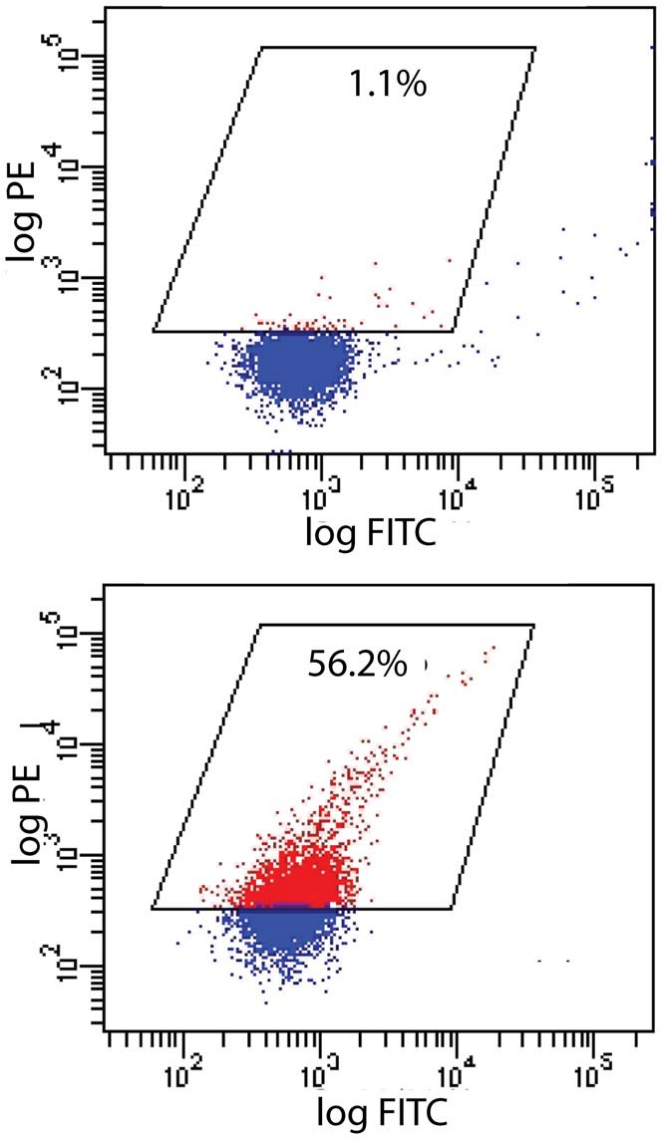
MACS/FACS results of MACS_545 Sample. Flow cytometry analysis of the fraction cell binding through conventional MACS plus FACS sorting after incubation with Streptavidin-Phycoerythrin (SAPE) labeled biotinylated PA protein target. The intensity of PE fluorescence (*y*-axis) represents either the expression of the surface peptide or affinity of the target to the display peptide. In the top dot plot, the cells are incubated with SAPE+biotin-PA prior to arabinose induction (negative control). The bottom dot plot shows the fraction PA bound after arabinose induction of the MACS_545 cells.

**Figure 6:**
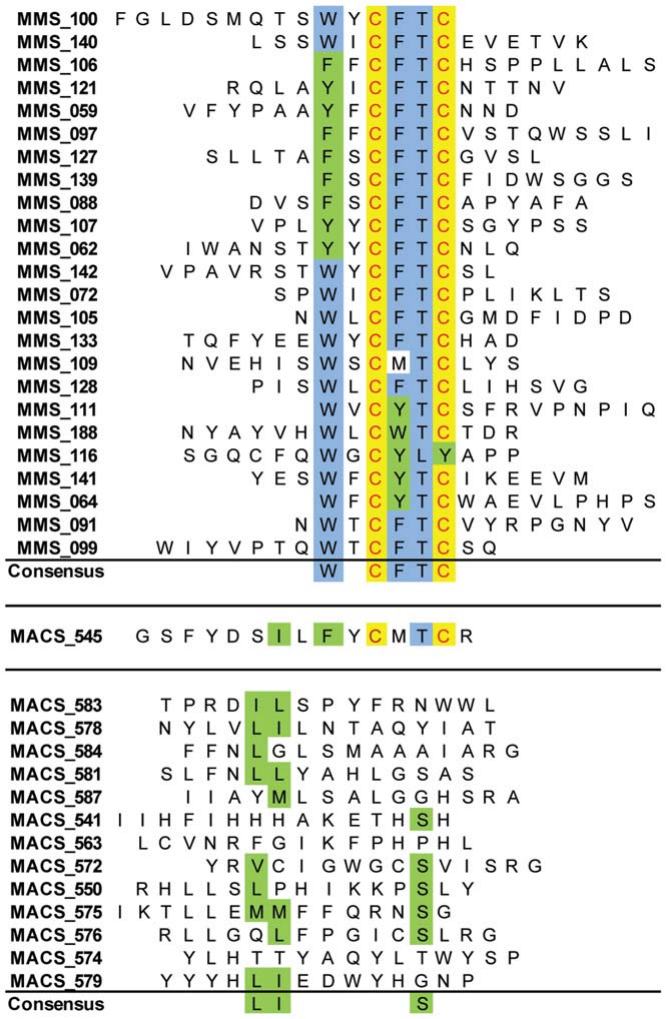
Sequence Alignment of MMS and MACS/FACS Sorts. Peptide sequences of clones selected by MMS system and MACS for binding to protective antigen. Conserved residues are highlighted in blue, similar residues in green, and identical residues in yellow. All of the twenty-four MMS selected sequences and one of the MACS sequences contained a six residue consensus sequence of WXCFTC.

For the evaluation of peptide binder selection by MMS and manual MACS from the peptide display library, the FACS analysis showed that after one round of MMS selection, the frequency of cells capable of binding to PA reached 0.7%, the second and third rounds further enriched the population to 56.1% and 65.5% respectively, with MMS selection (Figure 4), which is similar to the 56.4% seen for the MACS/FACS sort after two rounds of manual MACS and three rounds of FACS (Figure 5). Individual clones were picked at random from the positive populations and sequenced (Genewiz, South Plainfield, NJ). Sequences were analyzed and aligned using the Vector NTI (Invitrogen, Carlsbad, CA) software suite (Figure 6). Sequencing of 24 clones obtained after three rounds of sorting yielded 15 clones displaying a consensus motif **WXCFTC**. After repeating the selection process, a total of 24 distinct peptide sequences showed the **WXCFTC** consensus (Figure 6). This consensus was also found in one of the PA binders isolated the conventional MACS/FACS approach (Fig. 6).

Ten randomly selected MMS derived clones and fourteen MACS clones expressing peptides with the consensus sequences all show binding to 150 nM PA as measured by flow cytometry (Table 1). The MMS_128 had the greatest PA binding at 89.8% and only 0.2% for SAPE binding, which is greater than the best MACS/FACS sample, MACS_545 which had 62.0% PA binding and 16.3% SAPE binding. A number of the single clones analyzed for PA binding (Table 1) also show binding to Streptavidin (using SAPE), especially clones MMS_188, MACS_545, MACS_572, and MACS_575. Although not all of the MMS clones were tested, the amount of non-specific binding is greater in the MACS samples, with 10 of 14 clones having SAPE binding above 1.0% while only 1 of 10 MMS clones tested show SAPE binding greater than 1.0%.

**Table 1:** PA and Streptavidin binding analysis of Single Clones: The percentage binding from flow cytometry of ten of the MMS isolated clones and fourteen MACS/FACS isolated clones.

Clone	% Binding (150 nM PA)	% Binding (150 nM SAPE)	Clone	% Binding (150 nM PA)	% Binding (150 nM SAPE)
**MMS_140**	88.0	0.3	**MACS_583**	22.7	1.5
**MMS_127**	56.5	0.3	**MACS_578**	10.0	1.1
**MMS_142**	71.2	0.1	**MACS_584**	3.4	0.8
**MMS_105**	71.6	0.7	**MACS_581**	13.7	0.6
**MMS_133**	77.1	0.3	**MACS_587**	53.2	1.8
**MMS_109**	47.4	0.5	**MACS_541**	33.6	0.4
**MMS_128**	89.8	0.2	**MACS_563**	7.0	1.4
**MMS_111**	44.1	0.2	**MACS_572**	5.1	7.9
**MMS_188**	88.4	39.5	**MACS_550**	1.2	2.9
**MMS_141**	80.6	0.4	**MACS_575**	14.4	8.2
			**MACS_576**	3.9	0.6
**MACS_545**	62.0	16.3	**MACS_574**	23.6	3.1
			**MACS_579**	31.8	2.7

Each clone was tested against Dylight 488 labeled PA at 150 nM and Streptavidin-Phycoerythrin (SAPE) at 150 nM to assess the initial affinity to PA and the cross-reactivity to SAPE at 150 nM.

**Figure 7:**
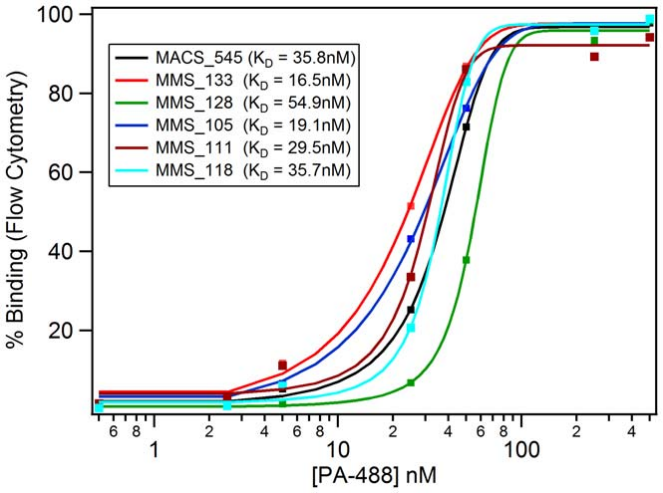
Affinity Analysis of Single Clones. Flow cytometry affinity analysis of the highest affinity clones for MMS and the best MACS/FACS clone. The cells were analyzed for affinity using 500, 250, 50, 25, 5, 2.5, 0.5, and 0 nM of the PA-Dylight 488 sample. The dissociation constant (K_D_) is shown for each sample.

In Figure 7, on-cell binding affinity analysis of 5 of the MMS clones with the comparable MACS/FACS sequence (MACS_545) was performed using dye-labeled PA (PA-dylight 488). The highest affinity PA binder was MACS_133 at 16.5 nM PA-488, which had twice the affinity of the best MACS/FACS binder (MACS_545 = 35.8 nM). Two other MMS clones tested showed higher binding affinity than the MACS/FACS sample, MMS_105 and MMS_111, which had affinities of 19.1 nM and 29.5 nM respectively. The MMS_128 sample had the lowest affinity of all clones tested, K_D_ = 54.9 nM.

## Discussion

A high throughput, semi-automated micromagnetic sorter (MMS) platform for bacterial display library sorting was introduced and described. Characterization of the MMS system's performance was achieved by screening against PA from *Bacillus anthracis*. Semi-automated magnetic activated cell sorters are not currently available for display library screening; therefore, results obtained using the MMS platform were directly compared to those obtained using conventional MACS/FACS sorting. In this work, we demonstrated that the semi-automated MMS platform is capable of effectively enriching affinity peptides against potential biological warfare agents with high throughput. For a typical 1 mL sample volume, MMS requires only 5 mins of user interaction, while manual selection requires more than 20 mins.

Three key parameters are used to evaluate cell sorting. The first parameter is *throughput*, which measures how many cells can be sorted per second. The MMS platform achieves high throughput screening since it is capable of screening a bacterial library containing 3×10^10^ members in 15 mins. With regard to gross throughput per hour, MMS is able to process 5×10^12^ cells/hr (50 mL/hr at a cell concentration of 1×10^11^ cells/mL), which is four orders of magnitude higher than that achieved using state-of-art FACS instrumentation or a previously reported dielectrophoretic cell sorter [29]. The surface area of the trapping region (Fig. 2) in the MMS disposable cartridge is a distinct advantage over manual MACS, which typically uses a 1.5 ml tube and benchtop magnetic for trapping. The trapping region in the MMS card is approximately 11.3 cm^2^ with magnetic trapping taking place on the top and bottom of the card (22.6 cm^2^ total area), while the 1.5 ml tube has a trapping region of approximately 3.2 cm^2^ on one side of the tube.

The remaining cell sorting evaluation parameters are *purity* and *recovery*. *Purity* describes the fraction of collected cells which actually bind target, and is evaluated using flow cytometry analysis of PA binder populations in the positive sample and the negative control sample as shown in Figures 4 and 5. The MMS displays comparable results to MACS/FACS for purity, with MMS having 65.1% PA binder population compared to 56.4% in MACS/FACS after 3 rounds of sorting (Figs. 4, 5). *Recovery* (the fraction of binders collected relative to the total number of binders in the naïve library) is assessed using a direct comparison of the MMS and MACS rare-cell and ultra-rare cell recovery results (Figure 3). Both magnetic sorting methods achieve ultra-rare cell recovery as shown by the sample population recovery down to 10^−8^. The MMS had greater overall total rare-cell recovery in the 0.1% and 0.01% (Fig. 3) samples compared to manual MACS, which is likely attributed to the greater surface area of the trapping region in the MMS card for accommodating a greater number of magnetic beads and cells. The manual MACS shows slightly better recovery at 10^−8^, which could be due to sample loss during the transfer from sample mixing tube to the syringe needed for MMS loading. For a more complete ultra-rare cell recovery at 10^−8^, a second round of cell-recovery following an overnight growth cycle would be beneficial for improved overall sample recovery for both the manual MACS and MMS.

Using the MMS, high affinity PA binders were isolated, which showed twice the affinity of the best MACS/FACS binder (Fig. 7). The isolated MMS clones also had lower overall binding to SAPE, 100-fold less binding on average, than the MACS clones (Table 1). This could be attributed to the use of the 1 µM biotin solution during PA selection, which seemed to decrease the streptavidin cross-reactivity in the MMS samples. Alternatively, the use of alternating SAPE and α-biotin PE FACS (during MACS/FACS analysis) selection did not seem to eliminate the SAPE cross-reactivity. Streptavidin clone enrichment in MACS and FACS is a problem when using streptavidin-coated magnetic beads [20], but the use of microfluidic sorting, such as MMS reported here and DACS results reported for epitope mapping [20], along with biotin additive to sorting seemingly decreases this unwanted enrichment The selection of PA binders and not SAPE binders was further evidenced with the PA on-cell binding affinity curves (Fig. 7) that used PA-488 instead of the PA-SAPE labeling for flow cytometry analysis. The results herein support the known issues with using streptavidin-biotin chemistry during selection; further studies with the use of biotin, SAPE, or α-biotin PE in MMS and MACS and FACS with bacterial peptide display libraries are warranted.

In addition to higher throughput, purity, and reproducible recoverability, for the first time, a PA binder sequence consensus WXCFTC was discovered and is corroborated with a PA binder selected by the classical MACS/FACS selection. Furthermore, the sorting protocols described here can be easily adapted to select other affinity reagents (yeast library comparison currently underway), by modifying the existing sorting methods in the LabVIEW interface, including, but not limited to, changing wash volumes, flow rates, and elution volumes.

The current results not only demonstrate the potential of the MMS platform for automated reagent discovery but could lead to a much broader extension to a variety of applications requiring rare-cell recovery for this or similar microfluidic technology. For example, the ability to consistently recover and isolate a rare cell population from a large negative control population provides a useful method for pathogen detection in food and water using this low cost, disposable cartridge system. The use of a disposable cartridge permits the analysis of potentially hazardous materials with minimal user exposure and eliminates any concerns for cross-contamination of samples. Above all, the MMS performs with consistency and can be coupled with display libraries to rapidly isolate peptide affinity binders for sensing, diagnosis, or detection of potential biohazard threats, such as protective antigen of *Bacillus anthracis*.

## Materials and Methods

### Micromagnetic sorter (MMS) System

#### MMS Disposable Microfluidic Card

The Micro-Magnetic Sorter (MMS) is an automated magnetic separation system consisting of a disposable microfluidic cartridge (Fig. 1a) and a companion instrument (Fig. 1b). The disposable cartridges are made of injection-molded polypropylene (Pinnacle Polymers PP 5135C). The 200 µm deep fluidic channels are defined by two injected parts, which are laser-welded (California Lasers, Simi Valley, CA) and a portion is heat staked with a hydrophobic membrane for bubble removal (Pall Co, Ann Arbor, MI) through the bubble trap (Fig. 1a). The trapping region (Fig. 1a) was designed to accommodate up to 1×10^9^ of 1 µm trapped magnetic beads and process up to 1×10^11^ bacterial cells. The trapping region has a total surface for binding of approximately 11.3 cm^2^ and total volume of 226 µl (11.3 cm^2^×0.200 cm depth). Female luer fittings on the top of the cartridge allow for a leak-proof interface between the cartridge and disposable syringes (Becton Dickinson, San José, CA). The luer fittings on the cartridge are designed to hold a reservoir array, for pneumatically driven applications as well as the injector inputs. There are a total of four luer ports required for two sample injectors (1 or 5 mL volume), one running/wash buffer injector (up to 10 mL) and one elution buffer injector (up to 3 mL volume). Strategically designed micro-channels allow for full automation of magnetic separation on the cartridge. To accomplish this, five pneumatically actuated pinch valves are located on the underside of the cartridge, which allow for the redirection of flow to one of the elution ports (Fig. 1a) during washing of unbound cells (to negative cell outlet) or elution of magnetically bound cells (positive cell outlet) (Fig. 2). These valve membranes require a force of ∼15 lb/in^2^ to seal and are robust enough to be actuated multiple times.

#### MMS Instrumentation

The instrument utilizes a cRIO controller with LabVIEW script (National Instruments, Austin, TX) outfitted with standard digital and analog in/out modules for control of the internal components. Flow rates within the cartridge are controlled by four stepper motors (Fig. 1c) and controller boards (Haydon and Anaheim Automation), which physically push on the injectors (Fig. 1d). These motors are fitted with micro-switches (Fig. 1c) (Panasonic ECG, Secaucus, NJ) that allow for the automatic calculation of input volume. Valves on the cartridge are actuated using pneumatically controlled air cylinders (SMC Corp, Noblesville, IN) and a DC diaphragm pump (Thomas provided by Nor Cal Controls, San Jose, CA). There are seventy custom neodymium-iron boron magnets, which are position-controlled by another Haydon stepper motor. The magnets are distributed equally among top and bottom portions of a magnetic rack, which sandwich the disposable cartridge. A single motor, in conjunction with a spring, allows for both horizontal and vertical movement of magnets. This facilitates horizontal movement required for trapping and elution, and vertical movement capable of agitating the sample within the cartridge.

### Cell Preparation for MMS Sorting

A bacterial display library (Cytomx Therapeutics; San Francisco, CA: eCPX library) which contains approximately 3×10^10^ members was screened for clones that display PA binding peptides The random library is first grown in 500 mL LB media containing 25 µg/mL chloramphenicol (LB-Cm^25^) to an OD_600 nm_ of approximately 0.6 (Eppendorf Biophotometer; Eppendorf, Hamburg, Germany) prior to induction by 0.04% (w/v) arabinose(the enhanced circularly permuted OmpX (eCPX) gene expressing the library peptides is under the control of an arabinose inducible promoter) [11]. The cells were shaken at 37°C for an additional 45 mins, after which the OD_600 nm_ was again measured and, using the assumption that an OD_600 nm_ of 1.0 relates to a bacterial concentration of 1×10^9^ cfu/mL, approximately 2×10^11^ cells were pelleted by centrifugation at 3000 g for 20 mins.

### Streptavidin-binder depletion

The bacterial pellet was re-suspended in 1.5 mL of PBSB (PBS buffer plus 0.5% BSA) containing 1×10^9^ paramagnetic beads (Invitrogen DynabeadsMyOneStreptavidin C-1; Invitrogen, Carlsbad, CA). The cell suspension was incubated at 4°C for 45 mins with rotation to allow depletion of streptavidin binders from the library prior to selections. To remove these beads and any cells bound to them, the sample was loaded onto an MMS cartridge and separated at a sample flow rate of 50 mL/hr and buffer flow rate of 10 mL/hr. The MMS cartridge captured the unwanted bead bound cells and allowed collection of the depleted library ready for enrichment. For the SA binder depletion using a benchtop magnetic bead separator (manual MACS), the bacterial cell pellet with 1×10^9^ paramagnetic beads was pelleted using a magnet next to the tube. The magnetic separation was performed for 5 mins to allow the bead pellet to form; the sample was washed and aspirated with 5×1 mL PBS washes, and re-suspended in 1 mL PBSB for PA binder enrichment.

### PA-binder enrichment

The SA-binder depleted library was centrifuged at 3000 g for 20 mins, re-suspended in 1 mL PBSB buffer containing 600 nM biotinylated protective antigen (EZ-Link Sulfo-NHS biotinylation kit; Thermo Scientific, Rockford, IL; List Biological Laboratories, Inc; Campbell, CA), and incubated at 4°C for 45 mins. Cells were centrifuged as above and re-suspended in 1 mL PBSB buffer with 1×10^9^ pre-washed magnetic beads. After 45 mins at 4°C with rotation, the cell-beads suspension was loaded into an MMS cartridge (or separated by manual MACS using the same methods as SA binder depletion). Bacterial cells bound to PA were trapped on cartridge, and then eluted into a 15 mL tube. A second round of sorting was performed following the same protocol as the first; however, the assay parameters were adjusted to account for the smaller starting population and to increase the selection pressure in the second round, therefore 1×10^8^ cells in 50 µL of 300 nM PA and 1×10^8^ magnetic beads were used. Cells were incubated static on ice for all labeling steps. Also, 1 µM biotin was added in the washing buffer to compete with any remaining streptavidin binders (peptides which bind to streptavidin typically have a much lower affinity than biotin). In the third round of MMS sorting, cells were labeled with 150 nM biotinylated PA, and then labeled with 1×10^6^ magnetic beads in 50 µL of PBSB. After each round of magnetic separation, the bead-bound enriched library was added to LB-Cm^25^ media supplemented with 0.2% glucose to inhibit expression of the eCPX gene and therefore prevent growth bias. The cultures were then grown overnight at 37°C with shaking.

### MACS/FACS Sorting

The optimized protocol was similar to previously published procedures [4], [12]. Briefly, The SA-binder depleted library was screened by FACS (BD FACSAria; BD Biosciences, Franklin Lakes, NJ), and PA binding clones were selected. This was repeated three times, alternating the secondary label between SAPE and an anti-biotin-PE antibody, which reduces enrichment of binders to the secondary label. After three rounds a highly enriched PA binding population was obtained. Binding was also assessed in the presence of BSA (Bovine Serum Albumin) and human IgG; binding to target was not reduced in the presence of these interferents, suggesting a specific interaction with PA.

### Analysis of PA binder enrichment by flow cytometry

To quantify the library enrichment of potential PA binders, flow cytometry analysis (BD FACSCanto II; BD Biosciences, Franklin Lakes, NJ) was performed using biotinylated PA labeled with alternating fluorescent secondary labels: streptavidin, R-phycoerythrin conjugate (SAPE; Invitrogen, Carlsbad, CA), anti-biotin-phycoerythrin (Miltenyi Biotec; Bergisch Gladbach, Germany), and Neutravidin, R-phycoerythrin conjugate (NAPE; Invitrogen, Carlsbad, CA), similar to previously published procedures [4], [12]. Following each round of PA selection, the arabinose induced cell population was incubated with 100 nM biotin-PA solution for 45 mins. The sample was centrifuged at 3000 g for 10 mins to remove unbound biotin-PA and was resuspended in a 25 µL solution of PBSB with secondary label concentration of 5 µg/mL and incubated for 45 mins at 4°C. The sample was centrifuged and resuspended in 1 mL ice-cold BD FACSFlow (BD Biosciences, Franlin Lakes, NJ) sheath immediately prior to flow cytometry. Cells labeled with SAPE exhibit increased red fluorescence and are easily distinguishable by flow cytometry.

### Affinity analysis of PA binders by flow cytometry

PA protein was labeled with Amine Reactive Dylight-488 NHS Ester dye molecule (Pierce Protein; Rockford, IL) according to the manufacturer's instructions. Briefly, a 1 mg sample of PA was dissolved into 2 mL of 0.1 M sodium carbonate pH = 8.5. The solution was added to a 50 µg sample of Dylight-488 NHS Ester for protein-dye conjugation. The protein-dye mixture was incubated for 1 hr at room temperature. The non-reacted, dye was removed by dialysis using a Slide-A-Lyzer 10 k MWCO membrane (Pierce Protein; Rockford, IL). To determine the on-cell binding affinity of each clone, the cells were grown and induced similar to Sorting Procedures and Sample Preparation mentioned above. A 25 µl PBSB solution of varying PA-Dylight 488 concentrations (500, 250, 50, 25, 5, 2.5, 0.5, 0 nM) was incubated with 5×10^6^ cells for 45 mins on ice. The samples were pelleted by centrifugation at 3000× g for 5 mins and resuspend in 500 µl BD FACSFlow for binding affinity analysis.

### Ultra-Rare Cell Recovery

To measure the rare and ultra-rare cell recovery of the MMS, negative control cells not expressing surface display peptides were doped with a known quantity of cells expressing a known PA binding sequence (MACS_545). A 1×10^−3^ or 0.1% PA binder (1 µL PA binding bacteria in 1 mL on negative control bacteria at identical OD_600_) sample was serially diluted using 1 mL of cells in 10 mL of negative control library to create samples ranging from 1×10^−4^ to 1×10^−8^ or 0.0000001% PA binding cells. The samples were analyzed by flow cytometry during four independent trials before and after MMS (or MACS) sorting to determine the ultra-rare cell recovery capability of each method.
